# AurkA inhibitors enhance the effects of B-RAF and MEK inhibitors in melanoma treatment

**DOI:** 10.1186/1479-5876-13-S1-P1

**Published:** 2015-01-15

**Authors:** Emilia Caputo, Roberta Miceli, Maria L Motti, Rosarita Taté, Federica Fratangelo, Gerardo Botti, Nicola Mozzillo, Maria V Carriero, Ernesta Cavalcanti, Giuseppe Palmieri, Gennaro Ciliberto, Giuseppe Pirozzi, Paolo A Ascierto

**Affiliations:** 1 Institute of Genetics and Biophysics –I.G.B., A. Buzzati-Traverso–, CNR, Naples, Italy; 2 Istituto Nazionale Tumori Fondazione G. Pascale, Naples, Italy; 3 Dipartimento di Scienze Motorie e del Benessere, Università di Napoli ‘Parthenope’, Naples, Italy; 4 Unit of Cancer Genetics, Institute of Biomolecular Chemistry (ICB-CNR), Sassari, Italy

## Background

Aurora Kinase A (AurkA), one of the key regulators of M phase progression, is over-expressed in melanoma and has been observed to limit tumor growth [1,2]. The potential use of this molecule as target for biological therapy in melanoma has been examined.

## Materials and methods

A375mel (BRAFV600E) melanoma cell line was used in this study. The cell line was exposed to B-RAF inhibitor (GSK2118436), MEK inhibitor (GSK1120212) and AurkA inhibitor (MLN8054) as single agents or in various combinations (B-RAF plus AurkA inhibitor, MEK plus AurkA inhibitor) or in triple combination (B-RAF plus MEK plus AurkA inhibitor).

The effects on the cell growth of drugs, used as single agents and as different combinations, were examined by the xCELLigence technology. Total protein extracts were examined for p53 and c-myc protein expression by Western Blot analysis. The drug’s efficacy was also tested by using a 3D-human melanoma skin reconstruction model.

## Results

A375 (BRAFV600E) melanoma cells treatment with AurkA inhibitors in combination with B-RAF and/or MEK inhibitors alone and/or with both B-RAF/MEK inhibitors, increased the anti-tumor efficacy of the drugs than given as single agents.

The AurkA inhibitors enhancing anti-melanoma effect on B-RAF and MEK inhibitors was furthermore confirmed in a 3D-human melanoma model, where it was restricted to a melanoma cell sub-population localized at epithelial/dermal junction site. However, S-100 and Ki-67 positively stained spindle-shaped cells were detected in the dermal stratum, suggesting the presence of alive and proliferating melanoma cells.

## Conclusions

These findings provide new prospects for melanoma research. For the first time, based on these results, it was observed that the triple combination treatment was more efficacious as anti-melanoma therapy. Interesting, the treatment was efficacious only on polygonal-shaped melanoma cells present at the epidermal/dermal junction site as small nests, while spindle-shaped melanoma cells present in the dermal stratum remained alive and proliferating. This finding suggested that these cells may account of the drug resistance and so be responsible of disease recurrence later on. Molecular characterization of these dermal cells may be critical for the development of novel therapeutic strategies.
